# Low-frequency pitch coding: relationships with speech-in-noise and music perception by pediatric populations with typical hearing and cochlear implants

**DOI:** 10.1007/s00405-023-08445-4

**Published:** 2024-01-09

**Authors:** Hilal Dinçer D’Alessandro, Maria Nicastri, Ginevra Portanova, Ilaria Giallini, Francesca Yoshie Russo, Giuseppe Magliulo, Antonio Greco, Patrizia Mancini

**Affiliations:** 1 Department of Audiology, Faculty of Health Sciences, Istanbul University-Cerrahpaşa, Istanbul, Turkey; 2 Department of Sense Organs, Sapienza University of Rome, Rome, Italy

**Keywords:** Cochlear implant, Children, Pitch perception, Temporal fine structure, Music perception, Speech perception

## Abstract

**Purpose:**

This study aimed to investigate the effects of low frequency (LF) pitch perception on speech-in-noise and music perception performance by children with cochlear implants (CIC) and typical hearing (THC). Moreover, the relationships between speech-in-noise and music perception as well as the effects of demographic and audiological factors on present research outcomes were studied.

**Methods:**

The sample consisted of 22 CIC and 20 THC (7–10 years). Harmonic intonation (HI) and disharmonic intonation (DI) tests were used to assess LF pitch perception. Speech perception in quiet (WRSq)/noise (WRSn + 10) were tested with the Italian bisyllabic words for pediatric populations. The Gordon test was used to evaluate music perception (rhythm, melody, harmony, and overall).

**Results:**

CIC/THC performance comparisons for LF pitch, speech-in-noise, and all music measures except harmony revealed statistically significant differences with large effect sizes. For the CI group, HI showed statistically significant correlations with melody discrimination. Melody/total Gordon scores were significantly correlated with WRSn + 10. For the overall group, HI/DI showed significant correlations with all music perception measures and WRSn + 10. Hearing thresholds showed significant effects on HI/DI scores. Hearing thresholds and WRSn + 10 scores were significantly correlated; both revealed significant effects on all music perception scores. CI age had significant effects on WRSn + 10, harmony, and total Gordon scores (p < 0.05).

**Conclusion:**

Such findings confirmed the significant effects of LF pitch perception on complex listening performance. Significant speech-in-noise and music perception correlations were as promising as results from recent studies indicating significant positive effects of music training on speech-in-noise recognition in CIC.

## Introduction

Advances in hearing technology such as development of newborn screening tools and cochlear implants (CI) allowed access to early diagnosis and effective intervention in children with bilateral severe-to-profound hearing loss. If intervened within sensitive periods, cochlear implanted children (CIC) are able to acquire age-appropriate spoken language and auditory-verbal communication skills [1]. However, significant limitations are still present in their speech-in-noise [2] and music perception in comparison to typically hearing children (THC) [3, 4]. On the one hand, such perceptual limitations might stem from abnormal development of auditory neural structures associated with sensorineural hearing loss [5]. On the other hand, CIs’ technological constraints negatively impact both place and temporal coding mechanisms, consequently leading to poor pitch resolution, in particular in the low-frequency (LF) domain [6].

Pitch has been known to be encoded by two neurophysiological mechanisms: place coding (spectral pitch) and phase locking (temporal pitch). The role of these mechanisms in conveying pitch information is proposed to be frequency sensitive. The transmission of high-frequency (HF) cues is dominated by place coding mechanism that is based on tonotopic excitation of nerve fibers. Instead, the transmission of LF pitch cues is mainly dominated by phase locking, namely a time-based mechanism that locks onto the Temporal Fine Structure (TFS) of the acoustic signal [7, 8].

LF pitch perception is believed to be a key factor for complex listening performance such as speech-in-noise and music listening. This notion mainly depends on the significant link between LF pitch perception and TFS coding that is shown to be dominant in the transmission of acoustic cues below 1000 Hz [9]. Conventional CI technology is designed to focus on the acoustic characteristics of spoken language by conveying mainly envelope cues, i.e., slow rate amplitude fluctuations in speech over time [10]. Even in the early 1990s, substantial evidence for the effectiveness of such an approach comes from the big majority of postlingually deafened adult CI users showing speech recognition scores above 80%, when open-set sentences without visual cues are presented under quiet listening conditions [11]. Conversely, CI technology mostly discards LF TFS cues that are characterized by rapid oscillations in speech and music signals. Indeed, LF TFS cues become of the essence for music listening and speech perception in the presence of noise, especially when the acoustic background is fluctuating, as it happens very often in everyday listening situations [9].

The main challenges of pitch, speech-in-noise, and music perception in CI users have been widely studied in the last decade [12, 13]. However, these studies are mainly conducted in adult CI users whereas pediatric results are considerably more limited [4, 12]. Moreover, pitch perception studies in pediatric CI populations are usually based on the assessment of their skills in the overall speech frequency spectrum including HF cues where recent CI technology is known to provide better representation of acoustic cues than those in the LF domain [10]. This fact partially depends on the limited number of pediatric assessment tools for LF pitch perception and complex listening conditions such as speech-in-noise and music perception [14]. Motivated by these reasons, the present study aimed to investigate the effects of LF pitch perception linked to TFS coding on speech-in-noise and music perception performance by CIC and THC. Furthermore, the relationships between speech-in-noise and music perception as well as the effects of demographic and audiological factors on present research outcomes were studied.

## Materials and methods

This study was carried out in accordance with the ethical requirements of the 1964 Declaration of Helsinki, its later amendments, and the existing legislation in Italy. The present protocol was approved by the local ethics committee of Sapienza University of Rome (Protocol no: 259/2020). All parents gave written informed consent for their child's study participance.

### Participants

Inclusion criteria for all the study participants regarded the followings: Italian spoken as the primary language in the family, absence of associated disorders, no attendance at formal music classes, and normal cognitive level (> 25 percentile at Raven's Colored Progressive Matrices-CPM) [15].

CI group consisted of 22 children (11 males, 11 females). The mean chronological age at the time of study enrollment was 8 years (SD = 1.23, ranging from 7 to 10 years). The group comprised of 10 unilateral and 12 bilateral CI listeners. The mean age at implantation and duration of CI use were 2 years (SD = 0.96, ranging from 0.98 to 4.17 years) and 6 years (SD = 0.87, ranging from 3.20 to 8.00 years), respectively. All CIC did not show any residual hearing (unaided hearing thresholds in both ears ≥ 85 dB HL at octave frequencies between 125 and 6000 Hz). The mean CI threshold was 29.21 dB HL (SD = 4.32, ranging from 20 to 35 dB HL). Five children were fitted with FS4 strategy (Med-El cochlear implants, Austria) whilst 13 children were fitted with Hi-Res120™ strategy (AB^®^ cochlear implants, USA) and 4 children with ACE™ strategy (Cochlear^®^ devices, Australia).

TH group consisted of 20 children (10 males, 10 females). The mean chronological age at the time of test was 8 years (SD = 1.09, ranging from 7 to 10 years). All the THC showed PTAs ≤ 15 dB HL in both ears (Mean = 9.2, SD = 3.58 ranging from 5 to 15 dB HL).

## Procedure

Testing was performed in a sound-treated room. Unaided hearing thresholds for a warble tone at octave frequencies from 125 to 6000 Hz were measured via an Aurical audiometer (Otometrics, Natus Medical SRL, Italy) and TDH39 headphones as were aided thresholds in the Sound Field (SF). SF tonal/speech, pitch and music stimuli were administered through an Acer P253-MG computer (Hscinchu City, Taiwan) and a Sony TA-FE 320R preamplifier (Tokyo, Japan) connected directly to a single Tangent EVO E5 loudspeaker (Herning, Denmark) at 0° azimuth/1 m distance from the participant’s head. Bilateral listeners were tested under the daily listening mode to avoid an acclimatization effect and longer test duration. Test orders were counterbalanced across children to minimize the effects of learning and fatigue. The overall test session did not last longer than 1 h.

### Pitch perception

Harmonic intonation (HI) and disharmonic intonation (DI) tests from the A§E psychoacoustic test suite [8, 16] were used to evaluate LF pitch perception skills in both CIC and THC. Both tests are based on a discrimination task between one intonating and one non-intonating complex tone. In both tests, the non-intonating sound is contrasted to an intonating sound. The non-intonating stimulus is a harmonic complex signal of an F0 at 200 Hz and its three higher harmonics presented at lower intensities (− 6 dB at 2F0, − 12 dB at 3F0, and − 18 dB at 4F0). In the HI test, the intonating sound is characterized by an F0 frequency sweep together with its harmonics [from NF0 to *N*(F0 + Δ*F*), *N* from 1 to 4]. In the DI test, the sweep happens only at F0 [F0 to F0 + Δ*F*] whilst the harmonics remain stable. This results in disharmonic intonation. For both tests, the linear sweep is introduced at 330 ms after the beginning of the signal and lasts 120 ms. Each stimulus lasts 600 ms and an interval of 500 ms is separating the two consecutive stimuli. White noise is added to the stimuli (SNR + 10.9 dB) in order to sound more natural to the listener and intensity roving (± 2 dB) is applied in order to control for the effects of loudness cues.

The stimuli were presented at 70 dB SPL and the participants were requested to respond if the two consecutive stimuli were the same or different. The test phase was always preceded by training to familiarize the children with the task.

The test starts at a Δ*F* of 41 Hz, ranging between 0 and 214 Hz where 0 Hz means no change between two signals and serves as an internal check to increase the test’s reliability. An adaptive staircase procedure is used to estimate the 50%-point on a listener’s psychometric curve by increasing the Δ*F* for an incorrect response and decreasing it for a correct one. If the Just Noticeable Difference (JND) could not be estimated within 100 trials, the JND is set to a value (220 Hz) above the maximum Δ*F*.

### Speech perception

Open-set speech recognition in quiet/noise was tested in SF with the standard Italian phonetically balanced bisyllabic words for pediatric populations [17]. Test lists were preceded by a practice list. The pre-recorded 20 items word lists were presented at 65 dB SPL in quiet and at + 10 dB fixed SNR.

### Music perception

The Gordon test [18] was used to evaluate music perception. The test assesses children’s music perception skills for rhythm, melody, and harmony. It consists of two consecutive phases: the training and test phases. At the training phase, children are familiarized with the test material and the task. They are presented with a model and three subsequent tracks showing how items’ rhythm, melody and harmony may vary in respect to the model. Finally, four pre-test trials are executed to ensure task understanding. The test phase consists of consecutive listening of the model and two test tracks named item 1 and item 2. After each item, they are asked to say whether item 1/item 2 is the same or different in respect to the model. This sequence of “model-item1-item2” is repeated 20 times. Hence, children respond to a total of 40 randomly administered items: 10 for rhythm, 10 for melody, 10 for harmony, and 10 neutral (track equal to the model). Every correct response corresponds to one point and the maximum score is 40 points. Normative values were collected from 1000 children aged between 7 and 12 years. The outcomes showed a mean score of 33.07 (SD = 4.02), with a reliability of 0.91 and a standard error of 1.2. The outcomes are divided into five performance categories: insufficient for scores equal/inferior to 23; sufficient between 24 and 26; satisfactory between 27 and 33; optimum between 34 and 37; excellent between 38 and 40.

### Statistical analysis

Data analysis was performed with the Statistical Package for Social Sciences 25.0 (SPSS, Chicago, IL, USA). Nonparametric statistical tests were used because the data were not normally distributed. Outcomes were reported as median and minimum–maximum values.

For group comparisons, the percentages of correct responses for speech perception were transformed to Rationalized Arcsine Units (RAUs) [19]. Between-group performance differences (pitch, speech, and music perception) were studied with the Mann–Whitney *U* test. The statistical significance level was set at 0.05. For multiple comparisons with Bonferroni correction, the cut-off level for *p* values were set at 0.025 (pitch and speech) or 0.012 (music), as appropriate. Effect sizes were computed with Rosenthal’s *r* effect size (small effect = 0.10–0.30, moderate effect = 0.30–0.50 and large effect ≥ 0.50) [20].

Spearman’s Rank Correlations (for CIC, THC, and overall group) were carried out to investigate the effects of LF pitch perception on speech and music perception as well as to evaluate the effects of chronological age and audiological outcomes (age at implantation, duration of CI use, SF, and bisyllabic word recognition score in quiet (WRSq) and at + 10 dB SNR (WRSn + 10). Only statistically significant correlations were reported in the Results (*p* ≤ 0.05).

## Results

**Fig. 1:**
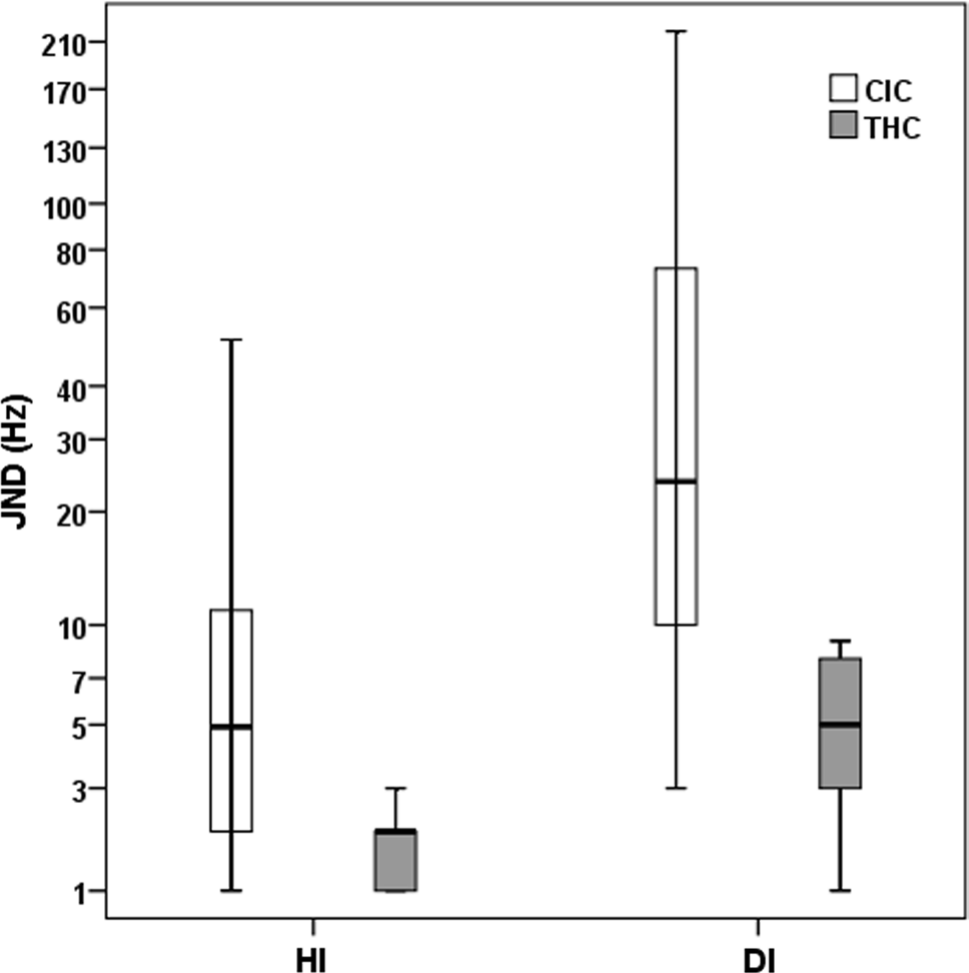
Pitch perception scores in CIC versus THC. *CIC* cochlear implanted children, *THC* typically hearing children, *HI* harmonic intonation, *DI* disharmonic intonation, *JND* just noticeable difference

**Fig. 2:**
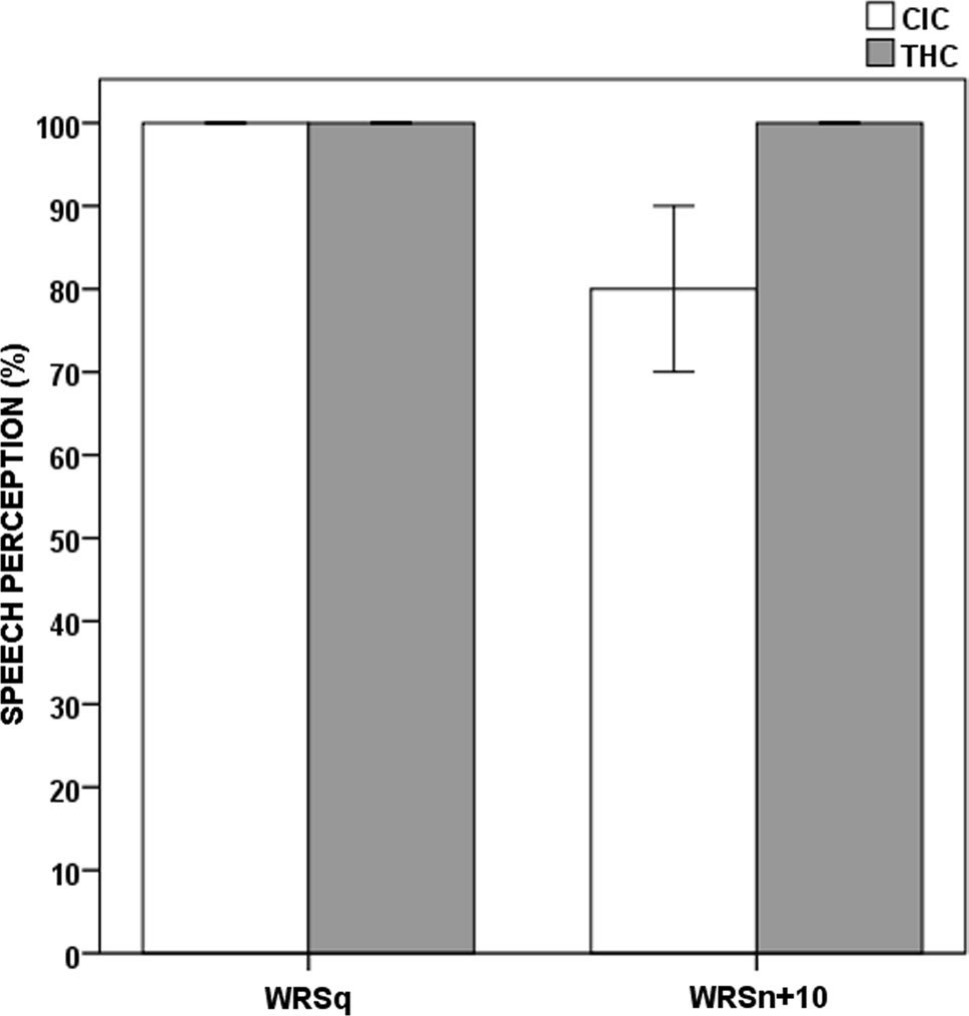
Speech perception scores in CIC versus THC. *CIC* Cochlear implanted children, *THC* typically hearing children, *WRSq* Word Recognition Score in quiet, *WRSn + 10* Word Recognition Score at + 10 dB signal-to-noise ratio

**Fig. 3:**
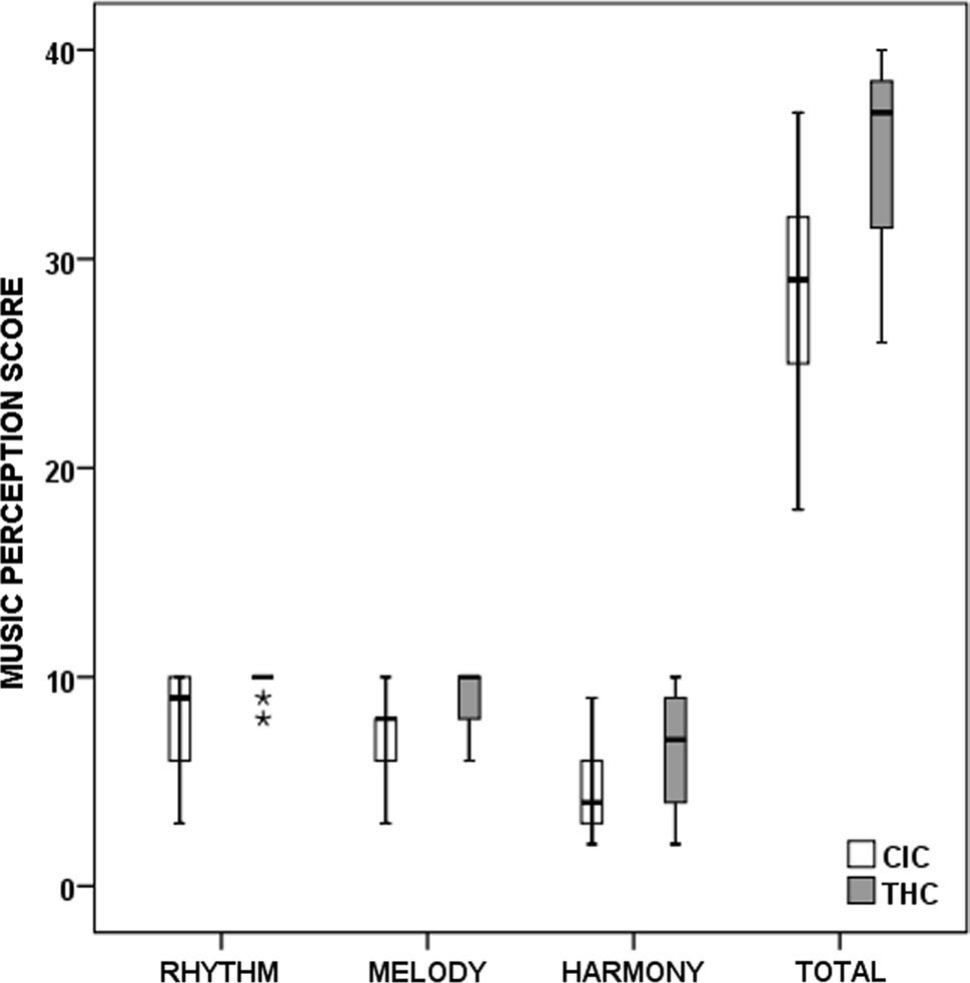
Music perception scores in CIC versus THC. *CIC* cochlear implanted children, *THC* typically hearing children

Pitch, speech, and music perception scores in CIC and THC are represented in Figs. 1, 2 and 3, respectively. The details of statistical findings are given in Table 1.

**Table 1:** Pitch, speech, and music perception outcomes in children with cochlear implants and typical hearing

	CIC		THC		Group Differences		
	Median	Range	Median	Range	U value	*p*	Rosenthal’s *r*
HI (Hz)	5	1–51	2	1–6	331.5	0.001*	0.51
DI (Hz)	24	3–220	5	1–9	376.0	< 0.001*	0.68
WRSq (%)	100	90–100	100	100	180	0.048	0.31
WRSn + 10 (%)	80	30–100	100	100	20	< 0.001*	0.84
Rhythm	9	3–10	10	8–10	117	0.002*	0.48
Melody	8	3–10	10	6–10	76	< 0.001*	0.58
Harmony	4	2–9	7	2–10	133.5	0.028	0.34
Total	29	18–37	37	26–40	77.0	< 0.001*	0.56

*CIC* cochlear implanted children, *THC* typically hearing children, *HI* harmonic intonation, *DI* disharmonic intonation, *WRSq* Word Recognition Score in Quiet, *WRSn + 10* Word Recognition Score at + 10 dB signal-to-noise ratio

*Statistically significant differences with Bonferroni correction

### Pitch perception

The median HI/DI JNDs in CIC were 5.0 (ranging from 1.0 to 51.0 Hz) versus 24.0 Hz (ranging from 3.0 to 220.0 Hz), respectively. The median HI/DI JNDs in THC were 2.0 (ranging from 1.0 to 6.0 Hz) versus 5.0 Hz (ranging from 1.0 to 9.0 Hz), respectively. Statistically significant group differences were observed for both HI and DI outcomes (*U* value = 331.50, *p* = 0.001 and U value = 376.00, *p* < 0.001, respectively).

### Speech perception

For the CI group, the median bisyllabic WRSq and WRSn + 10 were 100% (ranging from 90 to 100%) versus 80% (ranging from 30 to 100%), respectively. For both WRSq and WRSn + 10 listening conditions, the median score in the TH group was 100%, all children achieved the maximum score. Statistically significant group differences with Bonferroni correction were observed for WRSn + 10 (*U* value = 20.00, *p* < 0.001) whilst the differences for WRSq were not statistically significant (*U* value = 180.00, *p* = 0.048).

### Music perception

The details of music perception scores (median and minimum–maximum values) are given in Table 1. Statistically significant group differences with Bonferroni correction were observed for rhythm, melody, and total Gordon scores (*p* < 0.005) whilst the differences were not statistically significant for harmony discrimination (*p* > 0.05).

As per the performance categories reported by Gordon [18], CIC versus THC outcomes were as follows: insufficient scores (≤ 23) in 23% (*n* = 5) versus 0%, sufficient scores (ranging from 24 to 26) in 9% (*n* = 2) versus 10% (*n* = 2), satisfactory scores (ranging from 27 to 33) in 50% (*n* = 11) versus 25% (*n* = 5), optimum scores (ranging from 34 to 37) in 18% (*n* = 4) versus 20% (*n* = 4), and excellent scores (ranging from 38 to 40) in 0% versus 45% (*n* = 9).

### Correlational analysis

The details of correlational analysis resulting in statistical significance are given in Table 2.

**Table 2:** The correlations between pitch, speech, and music perception as well as the effects of demographic factors and audiological outcomes

	Age	CI Age	CI Use	PTA	HI	DI	WRSq	WRSn + 10	Rhythm	Melody	Harmony	Total
Age	**1.000**											
CI age		**1.000**										
CI use	0.663^**^		**1.000**									
PTA				**1.000**								
HI				0.534^**^	**1.000**							
DI				0.648^**^ 0.677^**^	0.548^*^ 0.507^**^	**1.000**						
WRSq						−0.323^*^	**1.000**					
WRSn + 10		**−** 0.445*		**−** 0.795^**^	**−** 0.454^**^	**−** 0.556^**^	0.350^*^	**1.000**				
Rhythm				**−** 0.494^**^	**−** 0.386^*^	**−** 0.367^*^	0.321^*^	0.385^*^	**1.000**			
Melody				**−** 0.589^**^	**−** 0.478^*^ **−** 0.596^**^ **−** 0.660^**^	**−** 0.462^**^		0.506^*^ 0.627^**^	0.429^*^ 0.523^*^ 0.608^**^	**1.000**		
Harmony		**−** 0.561^**^		**−** 0.400^**^	**−** 0.678^**^ **−** 0.416^**^	**−** 0.496^*^ **−** 0.437^**^		0.378^*^		0.472^*^ 0.691^**^ 0.624^**^	**1.000**	
Total	0.454^*^	**−** 0.530^*^		**−** 0.587^**^	**−** 0.652^**^ **−** 0.613^**^	**−** 0.480^*^ **−** 0.488^**^		0.458^*^ 0.577^*^*	0.567** 0.483* 0.644**	0.854** 0.745** 0.879**	0.579** 0.969** 0.807**	**1.000**

The correlations are in black for CIC and grey/italic for THC whilst those for the overall group are in bold

*CI* cochlear implant, *PTA* pure tone average, *HI* harmonic intonation, *DI* disharmonic intonation, *WRSq* Word Recognition Score in quiet, *WRSn + 10* Word Recognition Score at + 10 dB signal-to-noise ratio

*Correlation is significant at the 0.05 level, **correlation is significant at the 0.01 level

For the CI group, HI showed statistically significant correlations with melody discrimination (*r* = − 0.48, *p* = 0.024). Melody (*r* = 0.51, *p* = 0.016) and total Gordon scores (*r* = 0.46, *p* = 0.032) were significantly correlated with WRSn + 10. CI age had statistically significant effects on WRSn + 10 (*r* = − 0.45, *p* = 0.038), harmony (*r* = − 0.56, *p* = 0.007), and total Gordon scores (*r* = − 0.53, *p* = 0.011).

For the TH group, HI showed statistically significant correlations with melody (*r* = − 0.60, *p* = 0.007), harmony (*r *= − 0.68, *p* = 0.001), and total Gordon scores (*r* = − 0.65, *p* = 0.002). DI showed statistically significant correlations with harmony (*r* = − 0.50, *p* = 0.031), and total Gordon scores (*r* = − 0.48, *p* = 0.038). Hearing thresholds had statistically significant correlations with DI scores (*r* = 0.65, *p* = 0.003) whilst the chronological age had significant effects on the total Gordon scores (*r* = 0.45, *p* = 0.044).

For the overall group, both HI and DI showed statistically significant correlations with all music perception measures (ranging from − 0.37 to − 0.68, *p* < 0.05) and WRSn + 10 (− 0.45 and − 0.56, *p* < 0.01, respectively). Hearing thresholds showed significant effects on HI and DI outcomes (*r* = 0.53 and 0.68, *p* < 0.001, respectively). Hearing thresholds and WRSn + 10 scores were significantly correlated (*r* = − 0.80, *p* < 0.001), and both showed statistically significant effects on all music perception scores (SFs ranging from − 0.40 to − 0.60 versus WRSn + 10 ranging from 0.38 to 0.63, *p* < 0.05).

## Discussion

Speech recognition in the presence of noise and perception of music represent a great challenge for CI users. In particular, rich spectro-temporal cues of music (pitch, timbre, rhythm, melody, and harmony) are poorly transmitted by CI processing strategies developed primarily for speech recognition in quiet. Indeed, auditory perception through CI appears satisfying in allowing speech processing in quiet [11] but is still very limited in providing access to fine details of spectro-temporal variations in speech-in-noise and music [10]. Consistently with previous studies [12, 13, 21], present CIC versus THC performance comparisons for LF pitch, speech-in-noise, and all music measures (rhythm, melody, and overall) except harmony reveal statistically significant differences with large effect sizes whereas both groups seem to obtain excellent scores for speech perception in quiet (scores equal or above 90% even in all CIC). CIC performances for harmony are also considerably poorer than those of THC, but statistically significant differences disappear for this measure after Bonferroni correction. As per the performance categories reported by Gordon [18], only a very small percentage of CIC are able to obtain optimum scores whilst no CIC reveal excellent scores even in such a sample composed of remarkably good CI performers showing excellent word recognition in quiet.

To the best of our knowledge, the present study shows for the first time specifically the significant negative effects of limited LF pitch perception on speech-in-noise and music perception performance in pediatric populations, composed of both CIC and THC. Indeed, statistically significant correlations are not only observed between LF pitch, speech-in-noise, and music measures but also between speech-in-noise and music perception findings. The strength of correlations shows a considerable variability (weak to strong), with a trend towards stronger relationships of melody/total Gordon scores with HI and WRSn + 10. Such significant relationships are in line with previous findings from adult CI users [12, 13, 22] and as promising as those from recent studies showing significant positive effects of music training on speech-in-noise understanding [23].

Specifically for the CI group, statistically significant correlations are observed between melody perception and HI outcomes. These results are consistent with previous findings by Dincer D’Alessandro et al. [6] showing significant speech-in-noise correlations only for the HI test in adult CI alone listeners. Despite interindividual variability, DI JNDs are observed to be remarkably worse than HI scores where relatively higher frequency cues (still below 1000 Hz) are available to the listener due to the sweep of F0 together with its upper harmonics [16]. Thus, it might be suggested that CIC who could better use TFS information provided through some additional HF cues tend to show better speech understanding in noise. Conversely, the DI performance where the accurate perception of pitch stimuli is mainly dominated by time-based coding due to the sweep of only F0 (spectral information only below 300 Hz) is so poor in the majority of CIC that performance changes do not significantly interact. It is also worth noting that CIC findings show considerably better HI/DI scores compared to previous studies conducted in postlingually deafened adult CI users [6, 24]. On the one hand, such performance differences might result from small sample sizes of the existing studies. On the other hand, CIC might tend to perform better because of some neuro-audiological differences between pediatric and adult deaf populations. More precisely, CIC might benefit from a larger spiral ganglion neuron population [25]. Moreover, thanks to higher cortical plasticity [26], a congenitally impaired auditory system might be developing strategies to compensate for the poor LF pitch/TFS cues provided by the CI system. Yet, the neurophysiological mechanisms for integrating spectral and temporal pitch information remain unclear but humans exhibit holistic pitch perceptions by assigning an LF pitch to sounds with HF components, even when LF components are absent (the missing fundamental) [7]. Such interesting aspects need to be studied in pediatric and adult CI populations. Some evidence comes from recent studies reflecting considerably better speech-in-noise perception in prelingually deafened long-term CI users compared to postlingually deafened adult CI populations [27].

For the TH group, statistically significant HI/DI correlations appear for music perception scores whilst they disappear for speech recognition. Indeed, significant HI/DI correlations with music perception measures might be linked to the non-linguistic characteristics of both stimuli. Previous studies suggest that tests based on non-linguistic LF pitch stimuli may better estimate its significant role in music perception whilst tests with linguistic stimuli may better reflect its role in speech perception [28, 29]. Nevertheless, the significant effects of LF pitch perception on both speech-in-noise and music perception performance become significantly evident in the overall group similarly with a previous study conducted by Fowler et al. in adults [12]. Indeed, it is reasonable to expect such significant correlations to become even stronger in larger pediatric CI populations including bimodal users benefiting LF residual hearing and a hearing aid in the contralateral ear [6, 13].

As for the effects of demographic factors and audiological outcomes, present findings show a significant effect of age at implantation on both speech-in-noise and music perception even in a group of early implanted sample (≤ 3.5 years), reflecting the significant role of very early auditory experience in complex listening performance [26, 30]. On the other hand, the chronological age results in a significant impact on the total Gordon scores in THC, suggesting that the test outcomes should be interpreted with caution in younger children. Another factor seems to be the hearing thresholds. Not surprisingly, significant effects of hearing thresholds on LF pitch, speech-in-noise, and music perception performance are evident for the overall group including both CIC and THC. However, significant DI correlations with hearing thresholds in THC are surprising, since the test is performed at suprathreshold levels. Such findings might be related to the small sample size.

The present findings should be interpreted considering the effects of small sample size for both CIC and THC. Indeed, the impacts of sound coding strategy as well as performance differences between unilateral and bilateral CIC could not be studied as well. Moreover, the group of CIC was composed of good performers, all showing an excellent speech recognition in quiet (WRSq ≥ 90%) and the majority reflecting a considerably good speech-in-noise understanding (WRSn + 10 ≥ 70%) than that typically observed in CI populations [2]. However, not all CIC perform as good as the present sample. Hence, it can be expected that performances may significantly deteriorate in poorer performers. On the other hand, after about 30 years of CI experience all over the world, a growing population of CIC becomes adolescents and young adults. Their long-term performance has become a hot topic in recent CI research and present aspects remain to be studied in these populations.

## Conclusions

Complex listening performance such as speech-in-noise and music perception continues to be a big challenge for CI users. Indeed, present CIC/THC comparisons for speech recognition in noise and music perception show statistically significant performance differences with large effect sizes. Significant effects of age at implantation on both performances are observed even in a group of early implanted sample, reflecting the significant role of very early auditory experience in complex listening performance.

To our knowledge, this study reveals for the first time specifically the significant negative impacts of limited LF pitch perception on speech-in-noise and music perception performance in CIC. Moreover, present findings for significant speech-in-noise and music perception correlations were as promising as those from recent studies indicating significant positive effects of music training on speech-in-noise recognition in CIC. Future studies are needed to better understand the effects of demographic and audiological factors such as listening mode and sound coding on such complex listening performance in CIC.

## Data Availability

The datasets generated during and/or analyzed during the current study are available from the corresponding author on reasonable request.
